# The practice of speleology: What is its relationship with spatial abilities?

**DOI:** 10.1007/s10339-022-01075-4

**Published:** 2022-01-31

**Authors:** Veronica Muffato, Michela Zavagnin, Chiara Meneghetti

**Affiliations:** Department of General Psychology, University of Padova, via Venezia 8, Padova, Italy

**Keywords:** Speleology, Spatial ability, Wayfinding attitudes, Spatial descriptions

## Abstract

Given the evidence of motor and exploring activities being related to spatial abilities on different scales, the present study considers the case of speleology, a peculiar underground exploratory activity. The relation of this practice with spatial abilities was examined. The study compares a group of expert speleologists (18), a group with a reduced amount of experience in speleology (19 novice speleologists), and a group with a similar amount of practice but in the outdoors (19 experts mountaineers). Group differences will be investigated in terms of (i) small-scale spatial task performance (rotation-based and spatial working memory); (ii) large-scale environment learning (reproduced using verbal descriptions) asking participants to learn a path through a cave or up a mountain (in a counterbalanced order) and then to test their recall with true/false spatial questions and graphical representation tasks; and (iii) self-reports of wayfinding attitudes. The results of linear models showed that, after controlling for age, gender, years of education, and vocabulary scores, expert speleologists had greater mental rotation and perspective-taking abilities and less spatial anxiety than expert mountaineers, and the former performed the true/false questions better than the latter. It should be noted that participants who reported having guiding/path-finding experiences had greater accuracy in graphical representation performance and higher scores in attitude towards orientation. Overall, expertise in speleology is related to spatial abilities on different scales and might have a distinctive role in comparison with other motor practices, pointing to the potential value of examining speleology in the spatial cognition framework.

**Supplementary Information:**

The online version contains supplementary material available at 10.1007/s10339-022-01075-4.

## Introduction

### Spatial abilities on different scales

There are different scales of spatial abilities that can be distinguished between small and large scale. Small-scale spatial abilities can be defined as the skills needed to generate, retain, and modify abstract visual images (Lohman 1988). They are tested using paper and pencil tasks and comprise a set of distinct skills (see Hegarty and Waller 2005; Uttal et al. 2013). Among them there are the rotation abilities based either on object rotation, i.e. mentally turning objects, generally measured with a mental rotations test (Vandenberg and Kuse 1978), or on subject rotation, i.e. having to imagine adopting a different position in space (Hegarty and Waller 2004)—which can be measured with a perspective-taking task (Kozhevnikov and Hegarty 2001). Further, there are the basic mechanisms involved in spatial processing information, as in the case of our visuospatial working memory (VSWM), that we use to retain and process spatial information (Logie 1995). Studies have shown that VSWM is related to small-scale spatial abilities with evidence that it composes a distinct factor (Meneghetti et al. 2014; Muffato et al. 2020) or is part of a single whole spatial factor that includes both rotation and VSWM (Hegarty et al. 2006).

If, on the one hand, the small-scale spatial abilities refer to the abilities that require one to work and manage information on a circumscribed space smaller than person’s body, on the other hand, the spatial abilities can be of larger extent (i.e. the space extends beyond the human body) and can be learned by collecting information from a plurality of points of view (Montello 1993) as occurs in navigation. Navigation is a complex process by means of which we experience a space from an egocentric point of view, based on sensorimotor information about our position in space, self-to-object distances, and self-motion. Navigating gives us a chance to learn a series of landmarks, turns, and changes of direction, and to memorize a set of place-action associations (Montello 2005). This demands the involvement and integration of multiple aspects (Wolbers and Hegarty 2010), such as sensory cues, computational mechanisms, and spatial representations, or cognitive maps (Tolman 1948). The taxonomies distinguish between a locomotion component, in which our body movements are coordinated with the local and proximal surroundings, and a wayfinding component, which involves an efficient goal-directed and planned movement through an environment (Montello 2005). We can also distinguish between wayfinding conditions depending on whether or not the target location and the path to reach it are known. The challenge for an individual who knows where they want to go (even approximately) and needs to find a way to get there (path finding) is made more difficult if they have no prior knowledge of the environment they need to pass through (path search; Wiener et al. 2009). Large-scale environmental information can also be acquired from symbolic media such as spatial descriptions or maps, both of which are associated with mental representations that have spatial features (Picucci et al. 2013). One way to present a path is to describe it in terms of simulated moves in the environment using egocentric words (turn right, turn left) that present the path sequentially, step by step, generating what is called a route description (Taylor & Tversky 1992). The resulting mental representation has spatial features that can be assessed with various recall tasks: people can be asked to demonstrate the environmental information they have learned by drawing a map (spatially based recall) or by judging true/false sentences expressing spatial relations between landmarks encountered along the path (verbally based recall) (see Gyselinck and Meneghetti 2011, for a review). Their mental representations (as assessed with the different recall tasks) have much the same spatial properties as those derived from a navigation experience (Picucci et al. 2013), and there is evidence of the spatial abilities involved being similar (Meneghetti et al. 2011, 2016).

Spatial cognition research also considers self-reported wayfinding preferences and inclinations, i.e. preferences and attitudes related to movements and environment activities (generally assessed using questionnaires) that represent spatial personal dispositions (He and Hegarty 2020). Among others, wayfinding inclinations include people’s perceived sense of direction (Hegarty et al. 2002), preferred environment representation mode (Pazzaglia and Meneghetti 2017), pleasure in exploring places (Meneghetti et al. 2014), and spatial anxiety (Lawton 1994). Increasing attention is being paid to examining the above-mentioned wayfinding inclinations, which have been found related to small-scale abilities (Meneghetti et al. 2014, 2020). There is evidence that wayfinding inclinations (in terms of strong sense of direction, greater pleasure in exploring places, and low levels of spatial anxiety) comprise a separate factor related to small-scale spatial abilities (e.g. rotation abilities; Meneghetti et al. 2020) and to VSWM (Meneghetti et al. 2014).

Concerning the relationship between large-scale spatial abilities, small-scale abilities, and wayfinding inclinations, studies have shown that both rotation and VSWM are related to large-scale spatial abilities, such as environment learning, as an accurate spatial information recall from navigation learning (Allen et al. 1996; Fields and Shelton 2006; Hegarty et al. 2006; Kozhevnikov et al. 2006; Weisberg et al. 2014), and rotation and VSWM abilities can play a different role in navigation learning (Meneghetti et al. 2016). Furthermore, wayfinding inclinations are related to environment learning, such as navigation (Pazzaglia and Meneghetti 2017), and they concurrently work with small-scale spatial abilities and VSWM to support the relationship with environment learning accuracy (Meneghetti et al. 2021a, b; Pazzaglia et al. 2018).

In the spatial cognition domain, there is great interest in examining spatial abilities on different scales and related factors (both individual and environment based). One way to do so is to consider individuals who participate in motor and sport practices for evidence of the relation with spatial abilities (Voyer and Jansen 2017). Speleology provides a peculiar case of motor activity based on navigation practice. It would be interesting, given that no evidence was available, to examine whether this practice is associated with high spatial abilities, in terms of their small-scale cognitive abilities, environment-related representations, and wayfinding preferences.

### Speleology, exploring activity, and spatial abilities on different scales

#### Speleology

Speleology can be classified as a “sporting science”, as it involves physically exploring caves (speleologists are also known as “explorers” or “researchers”) and its ultimate purpose is to discover new underground paths, study them, and share the new knowledge (Cant 2006; Mattes 2015). The activity lies at the interface between outdoor adventure sports and citizen science (Mencarini et al. 2021). Like other adventure sports (such as climbing, trekking, or scuba diving), speleology involves moving in a wild and extended environment, taking risks and coping with the unexpected (Pike and Beames 2013). The element of citizen science (i.e. a scientific project in which citizens collect, analyse, or disseminate data) lies in that speleological expeditions are also organized for the purpose of exploring and charting new paths (mapping).

The practice of speleology is interesting because it involves navigation in a very particular environment. The underground has limited natural visibility; thus, the elements and reference points need to be identified with the aid of a headlamp that casts its beam only a few metres in front of the explorer. It also contains an irregular terrain and shapes, including narrow, irregular passages, a series of dolines of varying depth, and sumps and tunnels, and it presents natural and unusual physical elements as referent points (e.g. rocks, water, and soil; Mattes 2015; Perez 2015). To move within a cave, other than physical preparation, appropriate equipment, and stamina, physical movements are required in vertical (moving from top to bottom and vice versa) and horizontal (moving to the right and left and vice versa) planes, according to sumps, tunnels, and terrain orientation, alternating them as needed. This strong body and sensorimotor experience in limited visibility and irregular environments might be a condition that favours spatial abilities on different scales.

Speleologists’ wayfinding abilities can be put to the test as their destination is frequently a cave, the whereabouts of which they may know to some extent (drawing on their memory), or may need to discover (path finding). This can be made more difficult when they need to seek new or alternative routes due to finding unexpected obstacles or gaps along their planned path, or when exploring caves for the first time, or visiting new parts of known caves (path search; Wiener et al. 2009). So, such motor practice for these types of tasks seems to be associated with navigation and wayfinding (e.g. path finding and path search) abilities. Examining speleologists’ spatial abilities may add to this study’s understanding of the relationship between exploratory practice and small- and large-scale spatial abilities and wayfinding attitudes.

To our knowledge, no research focusing directly on the different types of spatial abilities in speleologists has been published to date. Studies in the psychological domain have mostly examined personality-related aspects, such as sensation seeking (Zarevski et al. 1998), or emotional traits (as depression, anxiety, and frustration; Congia et al. 1982; see McEwan et al. 2019 for a review), but not speleologists’ cognitive (spatial) abilities. Some inspiring evidence, presented in the next paragraph, came from studies examining how environment exploration practice (mostly outdoor) with sports or motor activities is associated with spatial abilities.

#### Exploring activity and spatial abilities on different scales

Concerning small-scale spatial cognitive abilities, it has been demonstrated that elite athletes’ cognitive abilities are superior to those of non-elite athletes (Scharfen and Memmert 2019) and people expert in a given sport have been found to outperform less expert practitioners on several spatial (small-scale) cognitive tasks (see Voyer and Jansen 2017 for a meta-analysis). This finding is endorsed by reports that motor and spatial abilities engage similar motor networks in the brain (Zacks 2008). The meta-analysis by Voyer and Jansen (2017) showed up to large effects of sports practitioners’ motor skills on small-scale spatial task performance, though the effect was moderated by several variables (such as the category of spatial task, the specific task, the type of stimulus, and the type of sport). There are also reports of athletes having a greater VSWM (as tested with the Corsi Blocks task) than non-athletes (Barhorst-Cates 2019). Some evidence points to a relationship between small-scale spatial abilities and sports involving outdoor navigation (e.g. orienteering and mountain climbing) that, even to a different extent, requires to find an effective way to reach an unknown destination. The orienteering practice has been found associated with good spatial small-scale abilities, such as mental rotation (Malinowski 2001; Schmidt et al. 2016), perspective-taking and spatial visualization abilities (i.e. the ability to manipulate spatial stimuli) (Roca-González et al. 2017), and also with VSWM (Notarnicola et al. 2012). Mountain climbers were shown to have a good visual memory (Whitaker et al. 2020). Overall, there is some encouraging evidence of exploration-based sports relating to small-scale spatial abilities (Malinowski 2001; Roca-González et al. 2017; Schmidt et al. 2016) and visuospatial cognitive aspects such as visual memory (Whitaker et al. 2020).

Concerning large-scale spatial abilities, some studies suggest that exploration-based sports are associated with a good performance in route planning and path-finding activities (Hacques et al. 2021). Examining environment learning using a path integration task, i.e. measuring participants’ ability to track and update their position during locomotion (Loomis et al. 1999), some studies found athletes better at updating their position than non-athletes (Bredin et al. 2005; Popov et al. 2013). Other evidence comes from climbers who have developed specific abilities to better approach the environmental demand to accomplish the task. Studies have shown that the climber’s level of experience was associated with the ability to recall and repeat a series of climbing holds (Boschker and Bakker 2002; Pezzulo et al. 2010) and to plan and remember sequences on the actions of a climbing path (Whitaker et al. 2020) encouraged to be developed under certain conditions (Seifert et al. 2015); in addition, there was an increase in climbing fluency with extensive practice (Seifert et al. 2018). According to the authors, this improvement is because their “route finding” skills have increased.

Other studies have shown that outdoor-exploring activities, such as trekking (Bondi et al. 2021) and orienteering (Meneghetti et al. 2021a, b) or orienteering-like activities (Munion et al. 2019), are related to small- and large-scale skills. In a study on orienteering, Meneghetti et al. (2021a, b) asked expert and novice orienteers (who mostly practiced in a mountainous terrain) and controls (who engaged in no physical leisure activities) to study a city map and then perform a pointing task. The results showed that orienteering experts performed better than the other two groups. Munion et al. (2019) asked second-year military academy cadets to locate targets in a wooded terrain after a day of training in the same area using other target locations. The results showed that they were better able to identify the targets correctly after the training, and males outperformed females. In another study, Bondi et al. (2021) examined mountain-trekking experts’ performance in visuospatial cognitive abilities in terms of large-scale reproduction (reporting the sequence of left or right turning points looking at a city map) and small-scale (mental rotation) tasks, other than visual (photo-recognition) task. The results showed that all trekking experts performed good in both small- and large-scale spatial tasks (range of average performance: 4–6.20; Max 7) in everyday conditions (low altitude). However, no inference on their refined task performance can be made given the lack of a control group (e.g. people with no trekking experience).

These findings suggest that exploratory outdoor activity (in most cases examining orienteering and climbing) is related to better exploratory activity efficacy (as seen with climbers, e.g. Seifert et al. 2015, 2018; Whitaker et al. 2020) and new environment learning (as seen with orienteers in new map learning; Meneghetti et al. 2021a, b) or the completion of new tasks in the same environment (Munion et al. 2019).

Hence, this study examines whether the practice of speleology, a motor activity navigation based in a very peculiar environment (i.e. irregular, dark, and with a natural but unusual point of reference), is associated with high spatial abilities on different scales (i.e. in terms of both small-scale spatial abilities and large-scale environment learning). This permits enlargement of knowledge in the spatial cognition field examining the relationship between exploratory activities, considering speleology practice, with small- and large-scale spatial abilities. At the same time, little is known about wayfinding attitudes in sport practice, but the few available reports concerning sports based on exploring showed that people with such practices (e.g. orienteering) have a greater perceived sense of direction and make more use of cardinal points by comparison with novice or control groups (Meneghetti et al. 2021a, b; Cornoldi et al. 2003). From here, the study’s focus is to better examine whether exploring skills, as practiced in speleology, are related to better wayfinding attitudes.

### Rationale of the study

The aim of the present study was to examine whether the practice of speleology is associated with small- and large-scale spatial abilities, and wayfinding attitudes. From here, the study’s focus is to better examine whether exploring skills, as practiced in speleology, are related to better wayfinding attitudes. Thus, a group of expert speleologists was identified with a cross-sectional design study and compared with individuals with less speleology experience or other kinds of motor experiences. Both groups of speleologists navigated in a karst region in the Pre-Alps areas of the Veneto and Lombardy (in northern Italy), where caves develop both horizontally and vertically (even to a depth of 1 km) and mainly differ for their years of speleology practice. The third group included people with many years of hiking experience in the mountains (that we have called expert mountaineers), a group that could be able to accomplish spatial tasks (Bondi et al. 2021) and with abilities to use navigational maps in a mountainous environment (Murakoshi and Higashi 2015). The experts in speleology and mountaineers have the same number of years of practice in the Pre-Alpine environment, but the former was underground and the latter was aboveground. It is important to bear in mind that expert speleologists and mountaineers differ in the following ways: they explore different environments (underground vs. aboveground) with varying degrees of visibility (dark vs. light—most of the excursions are made during the day), and there are various different landmarks and movements related to path finding. In speleology, the path to follow and find may extend more or less horizontally or vertically, and speleologists need to adapt their posture to the situation (e.g. walking upright in a chamber, climbing a chimney, crawling on all fours, or sliding along a tunnel) using natural elements (e.g. rocks and water) that need to be specifically found and recognized as useful. Contrary to mountain hiking, the path may rise more or less steeply, but mountaineers mainly remain upright as they move along it, using visible and distinctive natural elements (e.g. rivers and refuge huts) along a well-marked path (at least in the northern Italian Pre-Alps and Alps). Thus, although both speleologists and mountaineers explore paths and could have good spatial task performance, caving differs with regard to the type of environmental and exploration conditions. So, comparing them (i.e. expert speleologists and expert mountaineers) permits the examination of whether various types of environmental and related exploration are associated with similar or different spatial abilities on different scales and wayfinding inclinations.

To accomplish our aims, the three groups of participants: (i) performed small-scale (object-based and subject-based rotation) spatial and VSWM tasks; (ii) answered wayfinding questionnaires assessing their sense of direction, spatial anxiety, and pleasure in exploring places to obtain a profile of their spatial preferences (e.g. Meneghetti et al. 2021a, b); and (iii) learnt a new path through an environment. The large-scale environment was presented with verbal descriptions (route descriptions) of a path as seen from a person’s point of view, and related moves. Learning a spatial description produces a mental representation with spatial features resembling those obtained by navigating in an environment (Picucci et al. 2013; Meneghetti et al. 2011, 2016). One description presented a path through caves, the other a mountain path. The use of path description in two environments allows examination into what extent individuals with various types of environmental experience are able to mentally represent a path in different environments (i.e. as cave or mountain). In both cases, recall was assessed by means of: true/false sentences expressing the relationship between elements; and graphical representations of the path to capture the spatial properties of the respondents’ mental representations (Gyselinck and Meneghetti 2011). Participants’ spatial representations derived from the spatial descriptions of the two types of environment (underground and outdoors) were then compared in the three groups. We also assessed the strategies expert and novice speleologists preferred to use while caving (Macquet et al. 2012; Munion et al. 2019).

We expected to see the results outlined below.

(i) Spatial abilities (on the small scale): expert speleologists might have better spatial abilities than novices. This is supported by evidence that a larger experience—in comparison with a smaller one—in motor activity is associated with better small-scale spatial abilities such as rotation skills (Meneghetti et al., 2021a, b), and motor activities can be associated with a better VSWM (Barhorst-Cates 2019). As concerns the comparison between expert speleologists and mountaineers, a good performance in small-scale spatial tasks might be expected in both because mountaineering can benefit (small-scale) spatial abilities too (as shown by outdoor exploration activity, i.e. orienteering, e.g. Munion et al. 2019; Meneghetti et al. 2021a, b); however, differences between the two expert groups will be examined.
(ii) Spatial abilities (on the large scale): expert speleologists might have better environment learning skills than novices. This is because of the evidence that large outdoor motor experience—in comparison with smaller ones—is associated with better route planning and more effective movements (as shown by studies on climbers, Pezzulo et al. 2010; Seifert et al. 2018) and is associated with new environment learning as well (Meneghetti et al. 2021a, b). Based on the many years of experience acquired by expert speleologists and mountaineers, both groups should be able to learn a new environment better than novice speleologists. Differences between expert speleologists with expert mountaineers will also be explored. Further, differences between the groups will be examined as a function of the type of environment leant (cave vs mountain path), and the recall task administered (Meneghetti et al. 2021a, b).
(iii) Wayfinding attitudes: expert speleologists and mountaineers might have more functional wayfinding attitudes than novices (e.g. low spatial anxiety and high sense of direction) because of their greater experience of exploring compared to novice speleologists (Meneghetti et al. 2021a, b; Cornoldi et al. 2003). Differences between expert speleologists and mountaineers will be examined.
(iv) Strategies: expert speleologists may report making greater use of appropriate wayfinding strategies than novices (Macquet et al. 2012; Munion et al. 2019).

## Method

### Participants

Fifty-six people volunteered to take part in the study: 18 expert speleologists, with at least 9 years of experience (*mean (M)* = 250.11 months, SD = 155.96; 14 males); 19 novice speleologists with 1 to 24 months of experience (*M* = 11.74 months, SD = 8.16; 13 males); and 19 expert mountaineers with at least 9 years of practice (*M* = 226.00 months, SD = 128.14; 13 males). A power analysis run with the “pwr” library in R for linear models, and considering 18 coefficients in the models (see below), showed that 54 people were needed to obtain a power of 0.80, an effect size of 0.35, and a *p* of 0.05.

Although there are different numbers of males and females in each group, the proportion of males/females in each group did not differ, χ^2^(2) = 0.52, Cramer’s V = 0.10, *p* = 0.77. The two groups of speleologists differed significantly in terms of months of practice, *F*(2, 53) = 23.99, *η*^2^_p_ = 0.48, *p* < 0.001 (see means above), while the expert speleologists did not differ significantly on this variable from the expert mountaineers (*p* = 0.53). Concerning the intensity of activity, the three groups did not differ significantly from the others in terms of hours of practice a month, *F*(2,53) = 41.44, *p* = 0.25 (expert mountaineers: *M* hours = 26.47, SD = 15.78; novice speleologists:* M* = 20.58, SD = 10.53; and expert speleologists: *M* = 26.50, SD = 9.69). Further, from the analysis of the characteristics of the sample, it emerges that expert speleologists (18) all reported working as guides and/or being involved in cave finding (mapping), and some of the novices served as guides and/or were involved in cave finding (mapping; 10), while others did not act in any particular role (9). Some of the expert mountaineers worked as guides and/or in path finding (6) or reported not acting in any particular roles (13). Thus, the proportion of guide/path-finding experience differed in the three groups, χ2(2) = 18.93, Cramer’s V = 0.58, *p* < 0.001. Given the higher experience in guiding and path finding in expert speleologists and given the impact of active exploration (vs. passive) on environment representation (Chrastil and Warren 2013, 2015; Barhorst-Cates et al. 2020), the guide/path-finding experience will be considered as a factor and inserted in the following analyses together with the group.

The mean age of the participants in each group differed, *F*(2,53) = 4.41, *η*^2^_p_ = 0.14, *p* = 0.02, the novice speleologists being significantly younger (*M* = 33.63, SD = 13.17) than the other two groups (*p* = 0.05), while the expert speleologists (*M* = 46.39, SD = 10.32) and mountaineers (*M* = 42.53, SD = 16.08) did not differ significantly by age (*p* = 0.39). Given the age-related differences found between novice speleologists in the other two groups (expert speleologists and mountaineers) and the role of age per se in cognitive task performance (Craik and Bialystok 2006), age will be considered as a control variable in further analyses. Further, years of education and crystalized abilities (e.g. vocabulary test; Wechsler 1981) were taken into consideration in the analysis of the sample features because of their role in cognitive and spatial task performance with increasing age (Ardila et al. 2000; Vanetti and Allen, 1988). Therefore, it was ascertained that the three groups did not differ in years of education and in vocabulary test. The three groups’ years of education (i.e. number of years of schooling) was similar, *F* < 1, *p* = 0.98 (expert mountaineers *M* years = 13.21, SD = 3.74; novice speleologists: *M* = 13.42, SD = 3.95; and expert speleologists:* M* = 13.44, SD = 2.66), as well they were similar the performance in vocabulary test, *F* < 1, *p* = 0.63 (expert mountaineers:* M* = 48.63, SD = 10.40; novice speleologists: *M* = 46.00, SD = 8.51; and expert speleologists: *M* = 45.89, SD = 10.57).

No differences regarding years of education, vocabulary test (as a measure of crystalized ability), and gender proportions emerged between groups. Nonetheless, these variables will be inserted in the subsequent analyses as measures related to the individual (together with age).

### Materials

#### Spatial measures

##### Spatial tasks

*Corsi Blocks task*—backward version (adapted from Corsi 1972, Mammarella et al. 2008, reliability *r* = 0.74). The experimenter taps on increasingly long series of cube-shaped blocks (from 2 to 9) on a plain wooden board with 9 blocks standing on it, and the participant is asked to repeat the sequence in reverse order. The task terminates when participants fail to repeat two series of the same length (with the same number of blocks) or when they complete all the series. The score is the maximum number of blocks correctly recalled.

*Mental Rotations Test* (MRT, short version, from De Beni et al. 2014, adapted from Vandenberg and Kuse 1978). This includes 10 items, each comprising five 3D figures (combinations of cubes). The task consists in finding two out of four figures that match the target one, but in a rotated position (max time: 5 min). The internal consistency is good (*α* = 0.80; De Beni et al. 2014). One point is awarded for each item in which both the correct figures are identified (max: 10).

*Object Perspective-Taking task* (OPT, short version, from De Beni et al. 2014, adapted from Kozhevnikov and Hegarty 2001). This includes 6 items, each comprising a set of objects arranged in a specific layout. The task consists in the participant having to imagine being alongside one object, facing another, and pointing to a third. Answers are given by drawing an arrow from the centre towards the edge of a circle to indicate the direction of the target object (max time: 5 min). The mean of the absolute angle of error is calculated (maximum 180°). The internal consistency is good (*α* = 0.81; De Beni et al. 2014).

##### Wayfinding attitude questionnaires

Three questionnaires (from De Beni et al. 2014) were used. They have shown good psychometric properties in an Italian sample (*α* = 0.70–0.87).

*Sense of Direction and Spatial Representation scale* (SDSR; De Beni et al. 2014; adapted from Pazzaglia et al. 2000). There are 13 items measuring sense of direction and preference for a survey-based environment learning mode (e.g. “Do you think you have a good sense of direction?”), knowledge and use of cardinal points (e.g. “When you are outside, do you naturally identify cardinal directions?”), preference for a route- or landmark-based environment learning mode (e.g. “Think about how you orient yourself in different surroundings. Would you describe yourself as a person who orients him/herself by remembering routes connecting one landmark to another?”). Degrees of preference were expressed on a rating scale from 1 (not at all) to 5 (very much). A total score is calculated. The internal consistency is good (*α* = 0.81; De Beni et al. 2014).

*Spatial Anxiety scale* (De Beni et al. 2014; adapted from Lawton 1994). There are 8 items measuring how anxious respondents feel in environmental situations (e.g. “going to an appointment in an unfamiliar part of the city”). Degrees of preference are expressed on a rating scale from 1 (not at all) to 6 (very much). A total score is calculated. The internal consistency is good (*α* = 0.81; De Beni et al. 2014).

*Attitudes towards Orientation Tasks scale* (AtOT). There are 10 items, 5 regarding pleasure in exploring new places, and 5 for pleasure in going to known places. Degrees of pleasure are expressed on a rating scale from 1 (not at all) to 6 (very much), and the sum of the items is calculated after reversing the scores for pleasure in going to known places. The internal consistency is good (*α* = 0.81; De Beni et al. 2014).

##### Caving strategy questionnaire

This is an ad hoc scale that lists 16 strategies (elaborated by one of the co-authors with experience in speleology in collaboration with expert speleologists not included in the study sample) that can be used to orient oneself in a cave (e.g. “Study the map before entering to memorize the sequence of reference points”; for the complete list, see Supplementary materials; Table S1). The degree of strategy use was rated on a rating scale from 1 (not at all used) to 5 (very much used).

#### Spatial descriptions

Verbal descriptions of two realistic environments were prepared, one describing a route in a natural underground environment (called the cave path), the other a route in a natural outdoor environment (called the mountain path). Both descriptions presented the route using egocentric words (e.g. “left”, “right”), and contained some metric information (distances in metres). The descriptions contained the same number of words (292 and 293, respectively, in the Italian version), and each described 10 landmarks (e.g. a outcrop of mugo pine outside or a doline along the cave path; ladders or a witch’s rope bridge along the mountain path). The routes included the same number of segments (4 straight sections, and 2 turns), and 2 changes in altitude (descending 15 m and then coming back up again in the case of the cave path; climbing 20 m and then descending again for the mountain path). This is an excerpt from the first part of the description of the cave path: “The cave is located near the recently-renovated ‘dei Bei’ hut, where you can leave the car. Behind the hut there is a rock face, at the base of which is the entrance to the cave. It is difficult to access because there is a thick outcrop of mugo pine right in front of it, which obstructs the entrance. Once inside, you will have to continue on all fours for a few meters until you reach a doline 15 m deep. Descend it and go straight on, away from the entrance, to the narrow ‘broken bones’ passage, which is particularly difficult to pass due to the numerous protruding spikes of rock. Once you are through, you will find yourself in the ‘cactus chamber’, so called because of the unusual shape of the stalagmites in it”. An excerpt from the first part of the description of the mountain path reads: “The path starts to the right of the farmhouse, where there is a large, centuries-old beech. Take the dirt track, footpath No. 301 and continue for a few km. You will come to a rock face where the via ferrata begins. Use the metal ladders to climb vertically for about 20 m. Then you will be back on footpath No. 301, which is fairly flat. Go straight on until you come to the so-called witch’s rope bridge (a name probably associated with popular legends), which passes over a beautiful stream of crystal-clear water. Beyond the bridge, follow the footpath No. 301, which turns right, and after a while you will come to a large clearing used as a mountain pasture”. A pilot study with undergraduates ascertained the similar degree of recall of the two descriptions.

#### True/false spatial sentences

Twenty true/false sentences testing spatial relationships were prepared for each text. To give some examples, for the cave path one sentence was “To enter the ‘cactus chamber’ you will go through the narrow ‘broken bones’ passage” (true), and another was “Once you have entered the cave, you will first find the narrow ‘broken bones’ passage, and then the doline” (false); and for the mountain path one sentence was “The witch’s bridge is located beyond the ladders” (true), and another was “Walking along the footpath No. 301 route you will come first to a clearing and then to the witch’s bridge” (false). One point was awarded for each correct answer.

#### Graphical representation task

This involves placing landmarks, and reproducing the path (segments) and altitudes of the environment described on a blank sheet of A4 paper. For scoring purposes, we considered: landmarks placed in the correct order (0–10), correctly represented segments (0–6), and correctly represented altitudes (0–2). The sum of these scores gave us a measure of overall accuracy (0–18). Two judges scored the maps independently according to the above criteria and their scores correlated closely (*r* = 0.95), so the first judge’s scores were considered in the subsequent analyses.

### Procedure

After signing the consent form, participants were tested in two sessions, the first collective and the second individual, each lasting one hour. In the first session, participants completed a demographic questionnaire, the vocabulary test, and the cave strategy questionnaire (only for speleologists). Then, they performed the spatial tasks and answered the wayfinding attitude questionnaires (sMRT, sOPT, SDSR, SA, AtOT), presented in balanced order across participants. In the second session, they performed the Backward Corsi Blocks task and then listened twice to the description of a route through a cave or on a mountain path (balanced across participants). Pictures of the landmarks were projected in a screen when they were mentioned in the descriptions. Participants answered the true/false questions and completed the graphical representation task. Then, they listened twice to the other description, while looking at pictures of the landmarks, and they answered the true/false questions and completed the graphical representation tasks as for the first description.

## Results

All analyses were run using R (R Core Team 2020). Table 1 shows the means and standard deviations of all the variables in the three groups. Table 2 shows the correlations between the variables in the sample as a whole.

**Table 1:** Descriptive statistics for all measures in the three groups

	Expert mountaineers	Novice speleologists	Expert speleologists			
	M	SD	M	SD	M	SD
Age	42.53	16.08	33.63	13.17	46.39	10.32
Years of education	13.21	3.74	13.42	3.95	13.44	2.66
Vocabulary score (0–70)	48.63	10.40	46.00	8.51	45.89	10.57
Backward Corsi Blocks task (0–9)	7.37	1.50	8.21	2.46	7.56	1.95
Short Mental Rotation Test (0–10)	5.05	3.52	7.00	2.69	7.67	2.38
Short Object Perspective-Taking task (0–180° error)*	30.73	15.85	20.88	11.18	17.90	8.23
Sense of Direction and Spatial Representation scale (5–65)	42.58	8.63	40.47	9.69	45.29	9.72
Attitude Towards Orientation scale (10–60)	38.16	8.33	37.89	5.15	42.33	6.99
Spatial Anxiety scale (8–48)	18.95	5.53	14.63	3.45	12.72	3.89
Cave path—true/false questions (0–18)	14.89	2.88	16.00	2.49	16.50	2.09
Cave path—graphical representation (0–18)	11.05	3.94	13.42	2.97	14.33	3.74
Mountain path—true/false questions (0–20)	13.68	2.41	16.26	1.82	16.11	2.32
Mountain path—graphical representation (0–20)	10.68	3.11	13.16	3.35	13.78	2.60

^*^Given that the score expresses the degree of error, a high value means a large degree of errors (i.e. low performance)

**Table 2:** Correlations between variables in the sample as a whole

	1	2	3	4	5	6	7	8	9	10	11	12
1. Age												
2. Education	− 0.10											
3. Vocabulary test	− 0.05	**0.48**										
4. Backward Corsi Blocks task	− 0.17	0.01	− 0.02									
5. Short Mental Rotations Test	− 0.30	0.11	− 0.03	0.33								
6. Short Object Perspective-Taking task	0.19	− 0.16	0.12	− 0.16	− **0.48**							
7. Sense of Direction and Spatial Representation scale	0.09	− 0.20	0.04	0.07	0.33	− 0.12						
8. Attitude Towards Orientation scale	0.18	0.03	0.01	− 0.06	0.24	− 0.22	**0.62**					
9. Spatial Anxiety scale	0.01	− 0.18	− 0.14	− 0.03	− 0.27	0.22	− 0.19	**− 0.39**				
10. Cave path—true/false questions	**− 0.40**	**0.37**	0.11	0.10	0.30	**− 0.42**	0.10	0.27	**− 0.37**			
11. Cave path—graphical representation	**− 0.37**	**0.35**	0.24	0.04	**0.59**	**− 0.37**	0.16	0.32	− 0.27	**0.55**		
12. Mountain path—true/false questions	− 0.28	0.20	0.12	0.26	0.33	− 0.11	0.10	0.08	− 0.32	**0.36**	**0.40**	
13. Mountain path—graphical representation	**− 0.41**	0.33	0.29	0.26	**0.62**	**− 0.34**	0.14	0.12	**− 0.41**	**0.48**	**0.61**	**0.60**

*N* = 56. For |*r*|≥ 0.28, *p* < 0.05; for |*r*|≥ 0.36, *p* < 0.01, and for |*r*|≥ 0.46, *p* < 0.001 (the latter two in bold type). Significant correlations are in bold

### Spatial abilities (small-scale) and wayfinding attitude questionnaires: group differences

Linear models were run stepwise, considering the spatial tasks and wayfinding measures (backward Corsi Blocks, sMRT, sOPT, SDSR, SA, AtOT) as dependent variables. Age, gender, years of education, and vocabulary task values were entered in a baseline model (step 0) to examine the group effect after accounting for their role (given their impact on spatial learning; Borella et al. 2014; Pazzaglia et al. 2018). Then, group (expert mountaineers vs. novice speleologists vs. expert speleologists) and guiding/path-finding experience (given the different proportion of people with this experience in the groups and its role on spatial tasks performance; Chrastil and Warren 2013, 2015) were entered in step 1. Standardized betas, *p*, and *R*^2^ are shown in Table 3. For all models, the variance inflation factors revealed no significant multicollinearity (VIF ≤ 1.76).

**Table 3:** Standardized betas, *p*, and R^2^ for all models

*Predictors*	Backward Corsi Blocks		sMRT		sOPT		SDSR		AtOT		SA	
	*std.* β	*P*	*std.* β	*p*	*std.* β	*P*	*std.* β	*p*	*std.* β	*P*	*std.* β	*p*
Step 0												
Age	− 0.20	0.175	**− 0.43**	**0.001**	0.26	0.059	− 0.01	0.952	0.13	0.364	0.02	0.874
Gender	0.24	0.469	**1.13**	** < 0.001**	**− 0.65**	**0.040**	**0.64**	**0.047**	0.46	0.169	− 0.29	0.377
Years of education	0.03	0.861	0.25	0.067	**− 0.34**	**0.028**	− 0.21	0.178	0.10	0.548	− 0.19	0.251
Vocabulary test	− 0.02	0.914	− 0.05	0.696	0.23	0.127	0.21	0.169	0.02	0.902	− 0.08	0.612
Step 1												
Group 2–1	0.38	0.309	0.29	0.307	− 0.47	0.138	− 0.32	0.344	− 0.16	0.635	**− 0.84**	**0.009**
Group 3–2	− 0.13	0.752	0.50	0.106	− 0.36	0.292	0.35	0.333	0.25	0.495	− 0.32	0.341
Group 3–1	0.25	0.554	**0.79**	**0.018**	**− 0.82**	**0.025**	0.03	0.931	0.09	0.818	**− 1.16**	**0.002**
Guiding/Path-finding experience	− 0.21	0.563	0.12	0.649	− 0.16	0.606	0.45	0.177	**0.68**	**0.044**	− 0.15	0.612
*R*^*2*^	0.06	0.45	0.32	0.21	0.20	0.33						

*N* = 56. Group 1: expert mountaineers; group 2: novice speleologists; group 3: expert speleologists. sMRT = short Mental Rotations Test; sOPT = short Object Perspective-Taking scale; SDSR = Sense of Direction and Spatial Representation scale; AtOT = Attitude Towards Orientation scale; and SA = Spatial Anxiety scale. In bold the values corresponding the significant predictors

For the backward Corsi Blocks, no predictors were significant in either of the steps (step 0 Δ*R*^2^ = 0.04, step 1 Δ*R*^2^ = 0.02). For the sMRT, step 0 accounted for 33% of the variance, with age and gender emerging as significant predictors. Step 1 accounted for another 12% of the variance, with group emerging as a significant predictor, i.e. expert speleologists had better mental rotation abilities than expert mountaineers. For sOPT, step 0 accounted for 18% of the variance, with gender and years of education emerging as significant predictors, and step 1 for 14%, with group emerging as a significant predictor, i.e. expert speleologists had better perspective-taking ability than expert mountaineers. For SDSR, step 0 accounted for 14% of the variance, with gender emerging as significant predictor, and step 1 for 7% with no significant predictor. For AtOT, step 0 accounted for 7% of the variance with no significant predictor, and step 1 for 13% with guiding/path-finding experience emerging as significant predictor. For the Spatial Anxiety scale, step 0 accounted for 5% of the variance, but no predictor emerged as significant. Step 1 accounted for 28% of the variance, with group emerging as a significant predictor, i.e. both novice and expert speleologists had lower levels of spatial anxiety than expert mountaineers.

### Spatial description (large-scale reproduction): group differences

In the graphical representation task, the landmarks recalled (i.e. placed in their graphical representation, though not necessarily in the right position) were ≥ 83% and, in each description, they did not differ between the three groups (cave path, *χ*^2^(12) = 8.780, Cramer’s *V* = 0.28, *p* = 0.76, expert mountaineers 83%, novice speleologists 92%, and expert speleologists 82%; mountain path, *χ*^2^(7) = 4.61, Cramer’s *V* = 0.20, *p* = 0.88, expert mountaineers 89%, novice speleologists 90%, and expert speleologists 86%.

Linear models were run stepwise, considering the results obtained with the true/false questions and performance in the graphical representation task as dependent variables. Age, gender, years of education, vocabulary task value, and outcomes of the spatial tasks and wayfinding attitude questionnaires (backward Corsi Blocks, sMRT, sOPT, SDSR, AtOT, and SA) were entered in a baseline model (step 0). Then, group (expert mountaineers vs. novice speleologists vs. expert speleologists), guiding/path-finding experience, and type of environment (cave vs mountain) were entered in step 1. The group was inserted to accomplish the main aim of analysing performance in the three groups regarding environment learning tasks. Further, the guiding/path-finding experience was inserted given the different proportions of people involved in this activity in the groups. This is also motivated by the fact that active navigation in the environment relates to more refined environment representation favouring spatial task performance (Chrastil and Warren 2013, 2015; Barhorst-Cates et al. 2020). The type of environment was controlled given that it can also impact the mental representation (Belingard and Péruch 2000).

Then, the group × type of environment interaction was entered in step 2. Standardized betas, *p*, and R^2^ are shown in Table 4. For all models, the variance inflation factors revealed no significant multicollinearity (VIF values ≤ 1.62).

**Table 4:** Standardized betas, p, and R^2^ for all the models

*Predictors*	True/false questions		Graphical representation	
	*std.* β	*p*	*std.* β	*p*
Step 0				
Age	**− 0.33**	**0.001**	− 0.21	**0.017**
Gender	0.20	0.388	− 0.01	0.954
Year of education	**0.25**	**0.023**	0.10	0.284
Vocabulary test	− 0.03	0.790	0.17	0.064
Backward Corsi Blocks	0.11	0.235	− 0.00	0.960
Short Mental Rotations Task	− 0.01	0.923	0.48	** < 0.001**
Short Object Perspective-Taking task	− 0.06	0.559	− 0.02	0.847
Sense of Direction and Spatial Representation scale	0.05	0.685	− 0.08	0.435
Attitude Towards Orientation scale	0.08	0.502	0.12	0.241
Spatial Anxiety scale	**− 0.24**	**0.011**	− 0.09	0.296
Step 1				
Group 2–1	0.40	0.104	0.01	0.949
Group 3–2	0.31	0.190	0.32	0.135
Group 3–1	**0.71**	**0.017**	0.33	0.204
Type of environment (cave vs mountain path)	− 0.18	0.267	− 0.07	0.637
Guiding/path-finding experience	0.13	0.543	0.39	**0.041**
Step 2				
Group 2–1 × Type of environment	0.59	0.132	− 0.22	0.516
Group 3–2 × Type of environment	− 0.26	0.509	0.08	0.820
Group 3–1 × Type of environment	0.33	0.405	− 0.14	0.679
*R*^*2*^	0.40	0.53		

*N* = 112. Group 1: expert mountaineers; group 2: novice speleologists; and group 3: expert speleologists. In bold the values corresponding the significant predictors

For the true/false questions: step 0 accounted for 31% of the variance, with age, education, and spatial anxiety emerging as significant predictors; step 1 accounted for a further 7% of the variance, with group emerging as a significant predictor, as expert speleologists answered more accurately than expert mountaineers; and step 2 accounted for 2% and no interactions were significant.

For the graphical representation task: step 0 accounted for 47% of the variance, with age and mental rotation emerging as significant predictors; step 1 accounted for another 5% of the variance with the guiding/path-finding experience emerging as significant predictor; and step 2 accounted for 1% and no interactions were significant. As the graphical representation task included several measures (sequential order of landmarks, path segments, and altitude), further analyses were run separately for each measure as well. Step 1 accounted for 9% of the variance for accuracy in representing the path segments, with the group emerging as significant predictor—expert speleologists proving more accurate than novice speleologists (*β* = 0.51, *p* = 0.020), but not of expert mountaineers (*β* = 0.47, *p* = 0.079), and a marginal effect of guiding/path finding (*β* = 0.37, *p* = 0.058).

### Caving strategy questionnaire

ANOVAs were run to compare the novice and expert speleologists’ self-reported ratings of the use of strategies to orient themselves in caves (see descriptive statistics in Supplementary material, Table S1). The two groups differed (considering only *p* < 0.003 to be significant, given the multiple comparisons) in the following strategy item ratings: “Inside the cave, pay attention to which way the water is flowing” (*F*(1,35) = 7.88, *p* = 0.008, *η*^2^_p_ = 0.18), “Turn around to see how the passages will appear on the way back” (*F*(1,35) = 10.90, *p* = 0.002, *η*^2^_p_ = 0.24), that the experts judged more used than the novices; and “Be accompanied by someone who knows the cave” (*F*(1,35) = 30.29, *p* < 0.001, *η*^2^_p_ = 0.46), that the novices considered more used than the experts. No differences emerged regarding the other strategies (from *p* = 0.03 to *p* = 0.66). For the remaining items, the two groups did not differ (from *p* = 0.03 to *p* = 0.89).

## Discussion and conclusions

This study examined the relationship between spatial abilities on different scales (i.e. small and large scale) and wayfinding attitudes with motor practice, newly considering speleology, the practice of caving. There is already evidence of the relation of sport and motor practices with small-scale spatial abilities (Vojer and Jansen 2017) and some evidence of the relation with large-scale spatial abilities in terms of specific demands related to the motor practice (e.g. path finding in climbing; Seifert et al. 2015, 2018) and new environment learning (e.g. new map learning in orienteering; Meneghetti et al. 2021a, b).

The novelty of the study is to take a motor practice, such as speleology, into consideration. This is a navigation-based practice (as other sports and motor practices), but in a very peculiar environment. The underground is characterized by limited visibility, unusual points of reference and an irregularly shaped terrain involving movements in various planes. It should be noted that psychological studies have considered speleology in relation to personality and motivation factors (e.g. McEwan et al. 2019), but there was a lack of evidence about the role of cognitive (spatial) abilities in speleology. Examining this issue would offer a new chance to enlarge knowledge about the individual factors related to spatial abilities on different scales in the spatial cognition domain.

To accomplish this aim, expert speleologists with a great number of years of practice (at least 9 years) were compared with novice speleologists (i.e. a group with lower years of practice; up to 2 years for novice speleologists) and a group with the same great amount of years of practice (at least 9 years) in another navigation-based discipline (i.e. expert mountaineers). The latter discipline was selected because it is based on navigation experience, as in speleology, but in a different type of environment (outdoors) with various points (natural and visible elements) of reference and movements. The three groups were tested using: (i) object-based and subject-based rotation and VSWM tasks to measure their small-scale spatial abilities; (ii) true/false sentences and graphical representation tasks (after hearing paths described from a route view) to test their large-scale spatial abilities in recalling descriptions of a path through an environment. The three groups were also compared using: (iii) wayfinding questionnaires (sense of direction, spatial anxiety, and pleasure in exploring places). A caving strategy questionnaire was also administered to the two groups of speleologists.

The results concerning small-scale spatial abilities showed that expert speleologists performed better than expert mountaineers in the sMRT and sOPT, which test the rotation abilities, with the former required to mentally rotate objects and the latter to take different perspectives. This difference held after controlling for the effect of individual variables, among which gender was identified as a significant predictor for sMRT and sOPT, confirming its role in rotation task performance (Linn and Petersen 1985); in addition, age had an effect on sMRT performance (as already shown; Borella et al. 2014), and education had an effect on sOPT performance (as can happen in spatial task performance; Muffato et al. 2020).

As regards large-scale spatial abilities, our expert speleologists had a better spatial recall than the expert mountaineers in true/false questions and better than novice speleologists in path segment (straight sections and turns) in the graphical representations. Intriguingly, this difference between the groups was unrelated to the type of environment (cave or mountain), suggesting that experts speleologists form a better mental representation on hearing a description of a path, regardless of the setting. This result indicates that the years of experience in caves seem to favour path learning—at least verbally presented—in underground and aboveground contexts, assessed asking verbal judgments on elements’ location and their sequence. Therefore, it seems that the years of experience in caves facilitate the space representation that goes beyond the characteristics of the environment. This is surely interesting but must be better examined in further studies.

Here again, this difference between the groups persisted over and above any effects of individual variables, which were significant in the case of age (which predicted performance in both true/false questions and graphical representations), years of education (which predicted performance in the true/false questions) as seen for spatial and environment learning measures (Borella et al. 2014; Muffato et al. 2020). Additionally, spatial task performance and wayfinding questionnaire ratings have been taken into account because of the role of small-scale spatial abilities and wayfinding attitudes in environment learning, including when an environment is presented using verbal or written descriptions (Gyselinck and Meneghetti 2011; Meneghetti et al. 2011). When the role of these variables was examined, higher spatial anxiety ratings revealed a significant association with lower accuracy in answering the true/false questions showing that high spatial anxiety is negatively associated with spatial relationships judgment task performance. This might point to a specific role of spatial anxiety in environment learning task (Lawton 1994), or a state of anxiety prompted by the performance of a cognitive task in general (MacLeod 1996), and this needs to be disentangled in further studies. Further, it emerges that higher mental rotation scores also related with higher graphical representation accuracy, confirming that graphically reproducing a path learned from a verbal description demands spatial (rotation) abilities (Meneghetti et al. 2011). In short, spatial recall performance after learning from a verbal spatial description relate to individual variables (age and education), and individual spatial factors (spatial anxiety and mental rotation ability).

The answers given in the wayfinding attitude questionnaires indicate that only spatial anxiety differed between our three groups, with novice and expert speleologists reporting less spatial anxiety than mountaineers. As for the two speleology groups’ ratings of which strategies they preferred to use to orient themselves underground, the experts attributed more importance than the novices to such recommendations as “Inside the cave, pay attention to which way the water is flowing”, and “Turn around to see how the passages will appear on the way back”. The novices, on the other hand, judged it more important to be accompanied by someone who already knows the cave. These results newly extend to the field of speleology the relationship between specific strategies and certain motor practices (e.g. Macquet et al. 2012; Munion et al. 2019), an aspect that deserves to be better approached in further studies—also in relation to specific tasks required in exploring activities (Hacques et al. 2021).

In summary, our expert speleologists revealed greater rotation and perspective-taking abilities were better at memorizing an environment from a verbal description to solve questions testing spatial relationships and experienced less spatial anxiety (similarly to novice speleologists) than the expert mountaineers, while the only difference between experts and novice speleologists concerned the quality of their graphical representations when considering the accuracy in the path segments. It is noteworthy that the group of novice speleologists did not differ from the experts in the other comparisons, suggesting that about one year in mean of caving experience suffices to acquire much the same spatial abilities as expert speleologists. However, individual characteristics already present before engaging in the practice of speleology (e.g. personality and motivation factors; McEwan et al. 2019) could play a role in the choice of speleology practice and in turn relate to spatial abilities performance. For instance, personality and motivation-related aspects have been found to be related to small-scale spatial abilities (Moè et al. 2009), and both are related to better navigation performance (Pazzaglia et al. 2018). Therefore, individual characteristics related to speleology might prompt high spatial abilities. Although interesting, this is a speculation that must be better ascertained. The amount of experience in speleology, however, leads to a difference regarding some caving strategies, with the experts showing a more refined approach to use of strategies to orient themselves underground.

Overall, the present findings are consistent with reports that certain motor activities are associated with greater small-scale spatial abilities (Voyer and Jansen 2017), and this happens especially with regard to mental rotation and perspective-taking abilities (Schmidt et al. 2016; Roca-González et al. 2017). Furthermore, the findings are consistent with results showing that motor activities are also associated with stronger large-scale skills in terms of new environment learning (Meneghetti et al. 2021a, b), in this study reproduced with spatial descriptions, and with more functional wayfinding attitudes (Cornoldi et al. 2003), as revealed in the present case through lower spatial anxiety ratings. Specifically, this study found that the performance of speleologists but not mountaineers resembles, to a certain extent, the results found with motor activities involving the exploration of the outside environment, such as orienteering for both small- (Malinowski 2001; Schmidt et al. 2016) and large-scale abilities with new environment learning (Meneghetti et al. 2021a, b) and the relationship with wayfinding inclinations (as in Cornoldi et al. 2003).

However, the group is not the only factor that seems to have a role. The other factor emerging is guiding/path-finding experience. In fact, from initial analyses of sample features, it emerged that all of the expert speleologists served as guides and/or were involved in cave-finding activities, whereas the expert mountaineers did not seem to have an active role in finding the path, as most of them (13 out of 19) did not report to do so. Inserting guiding/path-finding experience as factor revealed that it predicts graphical representation performance (i.e. higher guiding/path-finding experience is associated with better graphical representation performance). This result is consistent with evidence showing that active navigation, such as walking, in comparison with being guided, is associated with a refined mental representation of an environment with map-view features (Chrastil and Warren 2013, 2015). Guiding/path-finding experience was also found to be related to the attitudes towards orientation, suggesting that this experience promotes the pleasure in exploring places, and this might contribute to defining the personal spatial profile (He and Hegarty 2020). However, after controlling for the role of the guiding/path-finding experience, the group effect emerged suggesting that the type of motor practice (being expert mountaineers or speleologists) could be important per se. At the same time, the investigation of spatial abilities in motor practice could be linked with other aspects, such as the type of exploration (active path finding) required with the practice.

In light of all the evidence, the results should be discussed in relation to the environment and request in speleology. Underground, natural elements to use to orient themselves need to be caught in the beam of the cavers’ headlamps and identified as appropriate. (A watercourse can be recognized as an element that helps to identify the correct path, for instance.) The path they take needs to be found as there are no markers and the terrain is usually very uneven (Mattes 2015; Perez 2015). The path may slope more or less steeply up or down, so speleologists can sometimes walk upright and sometimes have to crouch or crawl. Such different postures might enable them to develop mental representations of the environment with three-dimensional features (as suggested by spatial training based on promoting different body orientations to facilities such spatial representations; Liu et al. 2016). At the same time, caving is done in groups, where experts can have a more active role in guiding and finding routes compared to those with less experience. So, it seems that active guiding is part of speleology’s level of expertise. A great amount of experience in speleological activities seems to incorporate the guidance of others in caves and the exploration of new ways to reach a destination. Nevertheless, given the impact that active exploration can have on environment representation (Chrastil and Warren 2013, 2015) regardless of motor practice, further studies should attempt to consider these aspects (e.g. motor and guiding activities) separately. This is also motivated by the fact that not all navigation-based motor experiences require active guiding, as seen in this study’s expert mountaineer group, where few members reported having a guiding role. Also, for this difference between the expert groups, results showing elective spatial abilities in speleologists, although interesting, must be taken with caution, and future studies should disambiguate between the motor practice per se and the active path-finding experience.

So, this study is in line with previous evidence showing that practicing sports supports environment-exploring behaviours and strategies and tends to improve people’s route-finding skills (Hacques et al. 2021). However, this needs to be contextualized in relation to the type of motor activity that is being practiced and as a function of the specific demand in the context in which the activity is carried out. Speleology seems to be an interesting case of motor practice to develop spatial abilities on different scales. However, to reach clear conclusions that motor practice relates to spatial abilities, a longitudinal study design should be preferred over the current cross-sectional design. Studies on environment training and their benefits have offered insights. There is some evidence that training based on navigation in the environment produces benefits regarding recall tasks devoted to testing mental representations derived from learnt environment (conceptualized as near transfer effect; Lövdén et al. 2012; Ishikawa and Zhou 2020; McLaren-Gradinaru et al. 2020). Recently, for instance, Ishikawa and Zhou (2020) found that walking one route (once a week for 6 weeks) improved the accuracy of direction estimates. In navigation-based training studies, the effect on small-scale spatial abilities (conceptualized as far transfer effect) was not assessed. Some information on the far transfer effects of training on navigation-based activity came, again, from orienteering studies showing that orienteering training (i.e. based on exploration on large scale) improved participants’ small-scale spatial abilities (e.g. perspective-taking abilities Roca-González et al. 2017) and spatial working memory (Notarnicola et al. 2012). Framing these studies in the motor activity domain, the type of benefit stemming from motor practice is related to what extent motor activity produces benefits in specific (i.e. specifically related to the practice) and in general (as cognitive abilities) domains, and this represents an ongoing issue of debate (Scharfen and Memmert 2019; see Voss et al. 2010 for reviews and meta-analyses). Therefore, to disentangle to what extent a navigation-based motor practice (based on training) relates to large- and small-scale abilities (conceptualized as near and far transfer effects, respectively), a longitudinal study design must occur and then be considered in further studies. A longitudinal study design also allows to better control the individuals’ a priori features. In fact, individuals with already greater visuospatial abilities might choose to practice this type of exploratory activity or they might display predispositions and personality factors that support the choice of this practice (e.g. McEwan et al. 2019).

Another potentially relevant aspect concerns the use of tools and their metrics as maps in caving activities, given their specificity, i.e. how the environment is represented and features are depicted (cavers use ground plans with specific symbols to indicate elements such as water and rocks; Mattes 2015). The role of maps should be considered because of their role on learning from navigation (e.g. Meneghetti and Pazzaglia 2021), and landmarks have a role in the formation of a cognitive map (e.g. Epstein et al. 2017). At the same time, if verbal input is to be used in future studies, adding metric information to the environment description (only partially included in the current ones) might provide a better understanding of spatial representation characteristics (Noordzij and Postma, 2005) as a function of the type of motor experience.

Further, other aspects need to be examined to overcome the limitations of the present study, one of which concerns the types of group to compare. Expert mountaineers are certainly an interesting group due to their similarities and differences in comparison with speleologists (as reported above). However, the mountaineer group merits closer examination in terms of their small- and large-scale spatial abilities, given that they are in the condition to develop refined spatial skills with their practice, even controlling for motivation factors (Crust 2020) that can influence their choice to perform this activity. Other kinds of experts engaged in practices that involve path finding with movements in multiple environmental planes need to be considered, such as underwater practices where visibility is limited (e.g. scuba diving; Ergen et al. 2017) and air sport practices where there is a clear horizon (e.g. hang-gliding and parachute jumping; Buckley 2018). At the same time, an inexperienced group that engages in physical activities for leisure (e.g. 1–2 h a week) should be considered in comparison with a group with greater motor experience (Voyer and Jansen 2017). Another issue that deserves attention concerns individual spatial factors, such as: wayfinding attitudes—and spatial anxiety in particular—to elucidate how much they are specific or related to general personality dispositions (McEwan et al. 2019), and other types of small-scale spatial ability, since it has been shown that practicing motor activities is associated not only with mental rotation, but also with other small-scale spatial abilities (as spatial visualization, Roca-González et al. 2017) and spatial working memory (Notarnicola et al. 2012). Therefore, analysing these aspects can be important in clarifying the factors involved in the acquisition of spatial knowledge (Wolbers and Hegarty 2010) and will enable the practice of speleology to be better qualified in the spatial cognition domain.

To conclude, despite its above-mentioned limitations, this study offers preliminary evidence of the value of examining speleology, as the practice of exploring the underground environment, to examine the relation with spatial abilities on different scales, in the spatial cognition framework.

## Supplementary Information

Below is the link to the electronic supplementary material.

Supplementary file1 (DOCX 18 kb)

## Declarations

### Availability of data and material

On request to the main author.

### Code availability

On request to the main author.
