# Supplementary material for: The practice of speleology: What is its relationship with spatial abilities?
Source: Cogn Process. 2022 Jan 31;23(2):217–33. doi: 10.1007/s10339-022-01075-4 (PMC9072483; doi:10.1007/s10339-022-01075-4)
Supplement: Supplementary file 1 — Supplementary file1 (DOCX 18 kb) [file 10339_2022_1075_MOESM1_ESM.docx]

**Table S1.** Caving strategy questionnaire: descriptive statistics for the expert and novice speleologists.

| **Strategy** | Novices  *M (SD)* | Experts  *M (SD)* | F statistic |
| --- | --- | --- | --- |
| 1) Study the map before entering to memorize the sequence of reference points | 2.84 (1.46) | 3.39 (1.24) | *F*(1,35)=1.49, *p*=0.23 |
| 2) Before entering try to create a mental image of the map. | 2.89 (1.24) | 3.33 (1.24) | *F*(1,35)=1.16, *p*=0.29 |
| 3) Before you go, ask those who know the cave for information | 3.95 (1.27) | 4.11 (0.90) | *F*(1,35)=0.20, *p*=0.66 |
| 4) Be accompanied by someone who knows the cave | 4.84 (0.37) | 3.28 (1.18) | *F*(1,35)=30.29, *p*<0.001 |
| 5) Take the map into the cave | 2.11 (1.29) | 2.78 (1.35) | *F*(1,35)=2.40, *p*=0.13 |
| 6) Inside the cave, try to memorize the reference points | 4.37 (0.83) | 4.61 (0.50) | *F*(1,35)=1.14, *p*=0.29 |
| 7) Inside the cave, pay attention to which way the water is flowing | 3.21 (1.40) | 4.28 (0.83) | *F*(1,35)=7.88, *p*=0.008 |
| 8) Inside the cave, pay attention to air currents | 2.84 (1.17) | 3.78 (1.35) | *F*(1,35)=5.09, *p*=0.03 |
| 9) Turn around to see how the passages will appear on the way back | 3.42 (1.07) | 4.44 (0.78) | *F*(1,35)=10.9, *p*=0.002 |
| 10) Follow the footprints left by other speleologists | 2.79 (1.47) | 3.44 (0.86) | *F*(1,35)=2.69, *p*=0.11 |
| 11) In a vertical cave, refer to the depth of the wells | 3.47 (1.39) | 3.17 (1.25) | *F*(1,35)=0.50, *p*=0.49 |
| 12) Associate places with a name or an event to remember them better | 3.37 (1.16) | 2.67 (1.33) | *F*(1,35)=2.93, *p*=0.10 |
| 13) Place trail markers (e.g. stone men) to find the way back | 2.05 (1.39) | 2.56 (1.34) | *F*(1,35)=1.25, *p*=0.27 |
| 14) Follow the ropes or vie ferrate | 4.11 (1.24) | 3.72 (1.18) | *F*(1,35)=0.92, *p*=0.34 |
| 15) Note where rocks show signs of the passage of other people | 3.11 (1.33) | 3.72 (0.89) | *F*(1,35)=2.71, *p*=0.11 |
| 16) Follow directions (e.g. arrows in charcoal) | 3.42 (1.35) | 3.83 (0.86) | *F*(1,35)=1.22, *p*=0.28 |
